# Experimental and Field Data Support Range Expansion in an Allopolyploid Arabidopsis Owing to Parental Legacy of Heavy Metal Hyperaccumulation

**DOI:** 10.3389/fgene.2020.565854

**Published:** 2020-09-30

**Authors:** Timothy Paape, Reiko Akiyama, Teo Cereghetti, Yoshihiko Onda, Akira S. Hirao, Tanaka Kenta, Kentaro K. Shimizu

**Affiliations:** ^1^Department of Evolutionary Biology and Environmental Studies, University of Zurich, Zurich, Switzerland; ^2^Sugadaira Montane Research Center, University of Tsukuba, Tsukuba, Japan; ^3^Faculty of Symbiotic Systems Science, Fukushima University, Fukushima, Japan; ^4^Kihara Institute for Biological Research, Yokohama City University, Yokohama, Japan

**Keywords:** adaptation, expression ratio, heavy metal hyperaccumulation, homeolog, polyploid speciation, quantitative variation

## Abstract

Empirical evidence is limited on whether allopolyploid species combine or merge parental adaptations to broaden habitats. The allopolyploid *Arabidopsis kamchatica* is a hybrid of the two diploid parents *Arabidopsis halleri* and *Arabidopsis lyrata*. *A. halleri* is a facultative heavy metal hyperaccumulator, and may be found in cadmium (Cd) and zinc (Zn) contaminated environments, as well as non-contaminated environments. *A. lyrata* is considered non-tolerant to these metals, but can be found in serpentine habitats. Therefore, the parents have adaptation to different environments. Here, we measured heavy metals in soils from native populations of *A. kamchatica.* We found that soil Zn concentration of nearly half of the sampled 40 sites was higher than the critical toxicity level. Many of the sites were near human construction, suggesting adaptation of *A. kamchatica* to artificially contaminated soils. Over half of the *A. kamchatica* populations had >1,000 μg g^–1^ Zn in leaf tissues. Using hydroponic treatments, most genotypes accumulated >3,000 μg g^–1^ Zn, with high variability among them, indicating substantial genetic variation in heavy metal accumulation. Genes involved in heavy metal hyperaccumulation showed an expression bias in the *A. halleri*-derived homeolog in widely distributed plant genotypes. We also found that two populations were found growing on serpentine soils. These data suggest that *A. kamchatica* can inhabit a range of both natural and artificial soil environments with high levels of ions that either of the parents specializes and that it can accumulate varying amount of heavy metals. Our field and experimental data provide a compelling example of combining genetic toolkits for soil adaptations to expand the habitat of an allopolyploid species.

## Introduction

The ecological advantages of whole-genome duplication and its implication for species habitats have been debated for decades (Ohno, 1970; Stebbins, 1971; Comai, 2005; Soltis et al., 2014). Allopolyploids are interspecific hybrids of two or more parental species that inherit unreduced sets of chromosomes from their parental species. Allopolyploid species may have the potential to combine or merge parental adaptations (Doyle et al., 2008; Buggs et al., 2014; Huynh et al., 2020), which may provide enhanced abilities to tolerate extreme conditions (Levin, 2002; Adams, 2007). Inherited adaptations to abiotic conditions may also provide allopolyploids with the ability to inhabit areas beyond those of the parental species (Shimizu-Inatsugi et al., 2016; Van de Peer et al., 2017; Akiyama et al., 2019; Sun et al., 2020). The evidence of range expansion following allopolyploidization is largely circumstantial (Hoffmann, 2005; Blaine Marchant et al., 2016), therefore identifying ecologically relevant traits that may contribute to the broadening of habitats by allopolyploid species is essential (Ramsey and Ramsey, 2014).

The allotetraploid species *Arabidopsis kamchatica* has one of the broadest distributions of any *Arabidopsis* species (Hoffmann, 2005; Shimizu-Inatsugi et al., 2009) and offers a unique system to examine environmental responses at phenotypic and molecular levels. The species is a natural hybrid of the diploid parents *Arabidopsis halleri*, a hyperaccumulator of the heavy metals zinc (Zn) and cadmium (Cd), and *Arabidopsis lyrata*, a non-hyperaccumulator but with serpentine adaptation. Hyperaccumulating plant species, such as *A. halleri*, transport large amounts of toxic heavy metals (e.g., Zn and Cd) from the roots to the aerial parts of the plant and are also hyper-tolerant to high concentrations of these metals in soils (Bert et al., 2000; Pauwels et al., 2006; Krämer, 2010), whereas transport of heavy metals from roots to shoots is prevented in non-hyperaccumulators such as *A. lyrata* (Paape et al., 2016).

Metal concentrations in soils are an important factor for environmental niches, because high concentrations are toxic for most plants but hyperaccumulator species may survive there. The critical toxicity of soils for plants has been defined using arbitrary thresholds, for example, 100–300 μg g^–1^ for Zn and 6–8 μg g^–1^ for Cd (Krämer, 2010). Bert et al. (2002) proposed that soils with more than 300 μg g^–1^ Zn or 2 μg g^–1^ Cd be classified as metalliferous (or metal-contaminated) soils according to the French agricultural recommendation. Hyperaccumulator plants transport heavy metals from roots to shoots and can cope with high concentration of these heavy metals in soils. Hyperaccumulation in plants has been defined as the presence of >3,000 μg g^–1^ Zn and of >100 μg g^–1^ Cd in leaves (Krämer, 2010). *A. halleri* grows in both contaminated soils near mines and non-contaminated soils, and plants from both types of soils can hyperaccumulate heavy metals (Bert et al., 2002; Stein et al., 2017). Because hyperaccumulation is constitutive in *A. halleri*, this species may have evolved this characteristic as a mechanism to extract high amounts of heavy metals from metal-deficient soils for chemical defense (Boyd, 2007). Experimentally, *A. halleri* plants treated with Cd or Zn were more resistant to specialist and generalist insect herbivores (Kazemi-Dinan et al., 2014, 2015). The other diploid parent, *A. lyrata*, has known adaptations to serpentine soils, i.e., high concentration of magnesium (Mg) and nickel (Ni) in some of its regions of distribution. This appears to be local adaptation (Turner et al., 2010; Arnold et al., 2016) rather than a constitutive trait. While it is shown that some genotypes of *A. kamchatica* can accumulate substantial amounts of heavy metals under experimental conditions (Paape et al., 2016), the combination of laboratory data with those obtained *in natura* is necessary to study ecologically relevant natural variation (Shimizu et al., 2011; Yamasaki et al., 2017). Specifically, very little is known about whether *A. kamchatica* hyperaccumulates in field conditions, whether its habitats encompass soils with heavy-metal by natural or artificial processes, or how much quantitative variation in hyperaccumulation exists in the species.

The genetic basis of Zn and Cd hyperaccumulation has been studied extensively in *A. halleri* using comparative transcriptomics (Filatov et al., 2006; Talke et al., 2006), using QTL mapping in *A. halleri* and *A. lyrata* crosses (Courbot et al., 2007; Willems et al., 2007; Frérot et al., 2010) and functional genetics (Hanikenne et al., 2008). In *A. kamchatica*, these genes are inherited as homeologs from both diploid progenitors. The relative expression levels of both homeologs may be similar to the parental species (“parental legacy”), resulting in an expression bias (Buggs et al., 2014; Yoo et al., 2014) of genes involved in hyperaccumulation that were inherited from *A. halleri*. A bias in important heavy metal transporters among widespread genotypes of *A. kamchatica* would suggest that parental legacy or constitutive expression has been maintained throughout the species distribution.

Here we focus on heavy metal hyperaccumulation in *A. kamchatica* as an ecologically relevant quantitative trait, and aimed to answer the following questions. (1) Do the habitats of *A. kamchatica* contain high concentration of metals (Zn, Cd, Mg, and Ni) and is there evidence of artificially or naturally generated metalliferous soils? (2) Does hyperaccumulation of Zn and Cd occur in natural populations of *A. kamchatica*? (3) How much quantitative variation in hyperaccumulation exists in *A. kamchatica*? (4) Is *A. halleri* a more efficient hyperaccumulator than *A. kamchatica* at all concentrations of Zn? (5) Do genes involved in heavy metal transport and detoxification show a bias in the expression ratios of homeologs among *A. kamchatica* genotypes that may be derived from *A. halleri*? By combining field data with phenotyping in experimental conditions and homeolog-specific gene expression estimates, we provide the first range-wide study of constitutive heavy metal hyperaccumulation in an allopolyploid species.

## Materials and Methods

### Plant and Soil Material From Natural Populations

*Arabidopsis kamchatica* (Shimizu et al., 2005) is an allotetraploid species that is distributed in East Asia and North America. The diploid parental species *A. halleri* and *A. lyrata* each possess eight chromosomes (2*n* = 2*x* = 16) and the allopolyploid has 2*n* = 4*x* = 32 chromosomes. Al-Shehbaz and O’Kane (2002) reported that its habitats are gravelly slopes, forest, alpine regions, roadsides and flooded areas (under *Arabidopsis lyrata* subsp. *kamchatica* as a synonym). In contrast to *A. halleri*, it is not reported at sites with mining activities. Leaf tissues were collected from 40 *A. kamchatica* populations in Japan, Russia, and Alaska (United States), and soil samples from 38 corresponding locations were collected, from soil surface (0–1 cm depth) adjacent to a group of several *A. kamchatica* plants in each location, to quantify heavy metals in natural conditions (Supplementary Table 1). To identify sampling sites, we explored those natural populations by exploiting information on specimens that were available in museums and herbariums to cover a wide range of geographic regions, spanning altitudes from 30 to 2,949 m around three mountain chains in the Japanese Alps (Kenta et al., 2011). Additional samples were collected from Alaska, United States based on herbarium collection locations and previously reported sites (Shimizu-Inatsugi et al., 2009). For most populations, we collected leaf tissues from three or more plants. We reported values for leaf accumulation based on the mean and median of the replicates at each site. After visiting these natural populations, we noticed that some of the collection sites of *A. kamchatica* exhibited signs of human modification. Thus, populations were categorized into near construction or no construction as follows: (1) near construction populations are <10 m from buildings and fences on concrete base; or paved roads or riverside with concrete and/or asphalt, (2) no construction populations grew on native soil, free from concrete or asphalt or any obvious human modification. It is possible that populations not close to construction may have been cryptically altered by artificially occurring processes although it is not visible now. We also collected leaf tissues from *A. halleri* from two sites in Japan and two sites in Russia. Soil samples were collected from the two Japanese sites, which are known mine sites [Tada mine (TADA) and Omoidegawa (OMD), Japan; Briskine et al., 2017]. The Russian samples were collected from herbarium specimens; soils from these locations were not collected.

### Hydroponic Plant Growth Experiments

We conducted hydroponic experiments to measure Zn accumulation in leaves and roots using natural genotypes of *A. kamchatica* and *A. halleri* from germplasm collected. We used three, nine or 19 *A. kamchatica* genotypes. Two genotypes of *A. halleri* genotypes are TADA collected from a known mine site (subsp. *gemmifera*, a parental taxon of *A. kamchatica*), and BOD collected from a non-mine site (subsp. *halleri*). We also included synthesized *A. kamchatica* generated in our lab using *A. halleri* and *A. lyrata* parental genotypes (Akama et al., 2014), which is expected to possess identical or very similar sequences as the parents. Seeds of *A. kamchatica* were germinated on phytoagar (0.8%) and a mixture of oligonutrients (25 μM H_3_BO_3_, 5 μM MnCl_2_, 1 μM ZnSO_4_, 0.5 μM CuSO_4_, 50 μM KCl, and 0.1 μM Na_2_MoO_4_) and plated on a square (8 cm × 8 cm) petri dish until the seeds started to germinate (about 1 week). We used 1,000 μL pipet tip boxes (∼700 mL volume) as hydroponic chambers, so that seedlings could be grown in 0.5 mL thermo-PCR tubes using the 96-well insert (about 20 seedlings per box, for adequate spacing). The seedlings were then transplanted in 0.5 mL thermo-PCR tubes that were also filled with phytoagar solution and placed in the pipet tip boxes. The hydroponic solution was prepared according to Paape et al. (2016) and was composed of: 4 mM KNO_3_, 1.2 mM Ca(NO_3_)_2_, 0.8 mM MgSO_4_, 0.8 mM KH_2_PO_4_, 0.8 mM NH_4_Cl, and 5 μM Fe(III)EDTA. A separate 1 L stock of oligoelements was prepared with the following elements (16.25 mL of oligonutrients was added to the final 5 L solution): 0.2 mM KCl, 0.12 mM H_3_BO_3_, 0.04 mM MnSO_4_, 4 μM CuSO_4_, 1 μM ZnSO_4_, and 1 μM (NH_4_)_6_Mo7O_2_. All Zn treatments were in the form of ZnSO_4_ to ensure Zn is soluble in the hydroponic solution. Ten-liter batches were mixed in one container and then dispensed to individual hydroponic containers. The final pH was adjusted to 5.6–5.8.

To keep the moisture suitable for the growth of immature seedlings, a plastic bag was wrapped around each container to maintain a high level of humidity. The container was placed near natural light for 3–4 days. Plants that initially germinated but died in the immature stage were discarded. The final number of biological replicates for each accession varied from 5 to 13. Each hydroponic chamber contained a single plant genotype, to avoid root contamination between genotypes during sampling and harvesting. The boxes were then moved into a growth chamber (16 h light/8 h dark at 20°C) and the plastic bag was removed. The boxes containing the seedlings were placed on a 40 cm × 60 cm green tray (four boxes per tray) containing 1 cm of water and were covered with a plastic lid, to maintain a high level of humidity. The bottom of the plastic tube was cut with scissors after the roots grew to ∼0.5 cm, to allow the root to elongate into the box containing the hydroponic solution. A similar procedure was used for *A. halleri*, by placing freshly cut clones (i.e., ramets from a living plant) directly into the 0.5 mL tubes. Once the plants achieved the 3–4 leaf stage, the lid was removed to allow direct exposure to light. Zn supplements (Zn treatments added to the solution in the form of ZnSO_4_) were added after about 4.5 weeks of plant growth (with slight variability among genotypes because of seed germination). Leaves and roots were harvested from plants after 5.5 weeks of growth, with exposure to Zn treatment during the final week of growth.

We performed three Zn treatment experiments. In the first experiment, a supplement of 500 μM ZnSO_4_ was added to the hydroponic solution for 7 days. This experiment included 19 natural *A. kamchatica* genotypes and one synthetic polyploid that was generated from *A. halleri* subsp. *gemmifera* and *A. lyrata* subsp. *petraea* (Supplementary Material). We also used two naturally collected *A. halleri* genotypes that are maintained in our lab, TADA (*A. halleri* subsp. *gemmifera*, originally collected from the Tada mine, Japan) and BOD (*A. halleri* subsp. *halleri* collected from Boden, Switzerland), which can be clonally propagated for experiments. Because *A. kamchatica* is a self-fertilizing species, we can obtain offspring (seeds) from parents derived from single-seed descent. We grew 10–15 replicates per plant genotype, depending on germination.

The second experiment included nine *A. kamchatica* genotypes with a supplement of 1,000 μM Zn added to the hydroponic solution for 7 days. In the third experiment (“Zn gradient experiment”), Zn treatments were administered at 10-fold increments of Zn concentration: 1 μM (control condition) and 10, 100, and 1,000 μM, to compare relative accumulation at different concentrations and between species and genotypes. We tested three *A. kamchatica* genotypes selected to represent different geographic regions: Alaska (PAK), Japan (MUR), and Sakhalin Island (SAK), and two *A. halleri* genotypes, one from the Tada mine area in Japan (TADA) and another from Boden, Switzerland (BOD). For *A. kamchatica* we grew 10–12 replicates each, for *A. halleri* we grew 6–8 replicates of each genotype. Plants were grown for ∼4.5 weeks prior to the treatments. Leaves and roots were harvested after 48 h exposure to each of the four treatments, to measure the short-term uptake of Zn.

### Elemental Analysis in Plant Tissues and Soil Samples

Measurements of heavy metal concentrations were performed at the Institute of Terrestrial Ecosystems at ETH Zürich as described by Lahner et al. (2003) and Paape et al. (2016). For metal analysis in all three experiments, leaves and roots (approximately 2–5 mg dry weight) were harvested from plants after the Zn treatments. During harvesting, root tissues were washed in 150 mL of a cold solution of 5 mM CaCl_2_ and 1 mM MES-KOH (pH 5.7) for 30 min, followed by a wash in 150 mL of cold water for 3 min. The tissues were then collected in paper envelopes and dried at 60°C for 2 days. Root tissues were rinsed again with 18 MΩ water and placed into Pyrex digestion tubes. Leaf and root tissues were dried at room temperature and then at 50°C for 24 h.

Plant tissues were weighed and placed into 50 mL tubes. Samples were placed into an oven at 92°C to dry for 20 h prior to ion measurement. After cooling, reference samples for each ion were weighed. Samples were digested in a microwave oven with 2 mL of concentrated nitric acid (HNO_3_, ACS reagent; Sigma-Aldrich) and 30% hydrogen peroxide (Normapur; VWR Prolabo) and diluted to 10 mL with 18 MΩ water. Analytical blanks and standard reference material (WEPAL IPE 980) were digested together with plant samples. ICP-MS was used for the elemental analysis of samples and reference standards. To correct for instrumental drift, an internal standard containing yttrium and indium was added to the samples. All samples were normalized to calculate weights, as determined with a heuristic algorithm using the best-measured elements, the weights of the samples, and the elemental solution concentrations.

Soil samples were weighed and finely ground, then dried at 40°C. Soils were digested using the DigiPREP MS digestion system (SCP Science) and 2 M HNO_3_ for 90 min at 120°C. The samples were then cooled and diluted with up to 50 mL of nanopure water. The digested soils were filtered using Whatman filter paper into 50 mL centrifuge tubes. Samples were diluted and measurements were performed using inductively coupled plasma optical emission spectrometry (ICP-OES) at ETH Zürich.

### RNA Extraction and Pyrosequencing

Pyrosequencing of reverse-transcribed cDNA templates was used to measure the homeolog expression ratios of metal-ion transporter genes (see Supplementary Material for gene details). Pyrosequencing is a PCR-based method that can detect the relative abundance of homeolog-specific single-nucleotide polymorphisms (SNPs), which will vary according to homeolog expression levels (Akama et al., 2014). Leaf and root tissues from 15 *A. kamchatica* genotypes were harvested from three replicates grown in hydroponic solution at time zero (control) and at 48 h after the addition of 500 μM Zn (from the 500 μM Zn experiment described above). Leaf and root tissues were flash frozen in liquid nitrogen and stored at −80°C. RNA was extracted with TRIzol (Invitrogen) and purified using the RNeasy Mini Kit (Qiagen). RNA concentrations were measured using Nanodrop (Thermo Scientific). The RNA samples were reverse transcribed to cDNA using a High Capacity RNA-to-cDNA kit (Invitrogen).

Based on previous studies, we selected the orthologous *Arabidopsis thaliana* genes *HMA3* (AT4G30120), *HMA4* (AT2G19110), *MTP1* (AT2G46800), *MTP3* (AT3G58810), *NRAMP3* (AT2G23150), and *IRT3* (AT1G60960) (see Supplementary Table 8 for functional details and citations). We used coding sequence alignments in FASTA format that were generated from resequencing data of *A. kamchatica* homeologs (Paape et al., 2018). The PyroMark Assay Design v2.0 software (Qiagen) at the Genomic Diversity Center (GDC), ETH Zürich, was used to design PCR primers, sequencing primers, and SNP assays for each gene. To design pyrosequencing assays, we aligned the coding sequences of homeologous gene copies for each of six known heavy metal transporter genes in *A. thaliana* (*HMA3*, *HMA4*, *IRT3*, *MTP1*, *MTP3*, and *NRAMP3*), to detect SNPs between copies derived from *A. halleri* (H-origin) and *A. lyrata* (L-origin). We searched for target SNPs between two conserved primers that contained 2–3 target SNPs in regions <200 bp using the PyroMark software. The amplified PCR fragments were sequenced using PyroMark Q96 ID. Amplification peaks were analyzed using the allele quantification (AQ) mode in the PyroMark software to determine the SNP amplification ratio. The ratios of the 2–3 target SNPs obtained for each gene fragment were averaged to estimate the H- and L-origin homeolog expression ratios. Standard deviations for each gene were estimated from the three biological replicates. For the H-origin homeologs of *HMA4* and *MTP1*, we assumed that the H-origin expression was the sum of the duplicated copies derived from *A. halleri* (Paape et al., 2016).

### Statistical Analysis

For the leaf tissues and soils collected from natural populations, we calculated the mean, median, range, and standard deviations for each population, which typically consisted of three replicates. Correlations between leaf accumulation of Zn and Cd and soil concentrations of these heavy metals were assessed using Pearson’s correlation coefficients. We used ANOVA and linear models (lm) to detect quantitative variation in Zn accumulation in leaf and root tissues among genotypes of *A. kamchatica* in the 500 μM Zn-treatment experiment. We estimated broad sense heritability (*H*^2^) using the ANOVA table sum of square values to quantify between genotype variance relative to within genotype variance plus residual variance. We assessed whether the leaf and root Zn levels and the leaf/root ratio of Zn varied among genotypes of *A. kamchatica*. We performed this analysis with and without the outlier genotype, MAG, to determine whether trait variation was affected. We used the linear model formula: Zn ∼ species + genotype (separately for leaf, and for root) as a statistical test to determine whether Zn accumulation was significantly different between *A. kamchatica* and *A. halleri*. For the Zn gradient experiment, we compared the means and standard deviations of three replicates of each genotype at each Zn treatment condition.

We used linear models to determine the effects of genotype, gene (function), treatment, and tissue on homeolog expression ratios (estimated using pyrosequencing). The expression ratios varied from 0 to 1 according to the relative expression of either homeolog. If there was equal expression of both homeologs, then H-origin = 0.5 and L-origin = 0.5. H-origin ratio is H-origin/(H-origin + L-origin). Therefore, we used the expression of the H-origin copy as the dependent variable. The following linear model formula, which included both tissue types, was used: H-origin ratio ∼ genotype + gene + treatment + tissue + genotype × gene (interaction term) + gene × treatment (interaction term) + gene × tissue (interaction term) + gene × treatment × tissue (interaction term). The significance of each explanatory variable was summarized by the sum of squares and *F*-statistics in an ANOVA table. All analyses were performed in R, version 3.4.

## Results

### Zn Accumulation in Soils and Leaf Tissues in Wild Populations

**FIGURE 1:**
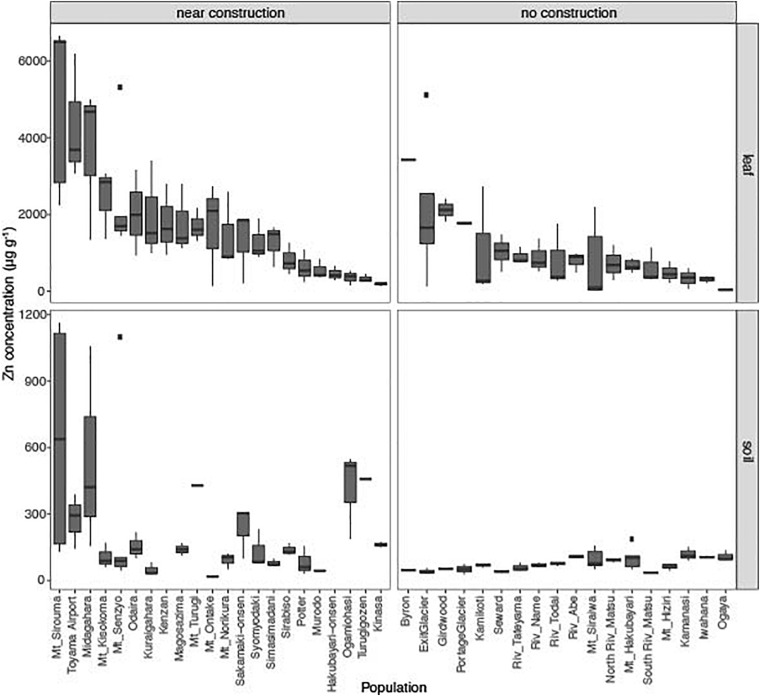
Leaf concentrations of zinc (Zn) from 40 natural populations of *A. kamchatica* **(Upper)**. Soil concentrations of Zn **(Lower)** at 38 corresponding sites. Populations were grouped into near construction or no construction types. The populations are sorted from highest to lowest Zn concentration in the leaf samples. The *y*-axis is the Zn concentration in μg per gram of dry weight. Note different *y*-axis scales for leaf and soil samples. Boxplots: center line, median; box limits, upper and lower quartiles; whiskers, 1.5 × interquartile range; points, outliers. The correlations between leaf accumulation and soil concentrations: Pearson’s *r* = 0.68 (near construction), Pearson’s *r* = 0.007 (no construction) (see also Supplementary Figure 1). Refer to Supplementary Table 1 for mean values, variance, number of replicates, and location information.

We searched for populations of *A. kamchatica* in Japan and Alaska (United States) by using herbaria and database information (Shimizu-Inatsugi et al., 2009; Kenta et al., 2011). We sampled leaf tissues from 40 populations of *A. kamchatica* and we obtained soils from 38 of these localities (Supplementary Tables 1–4). The concentration of Zn in these soils ranged from 18.7 to 642.8 μg g^–1^ (average, 153.3 μg g^–1^; Figure 1 and Supplementary Tables 1, 2). Although we found no population close to active or historical mine sites with heavy metal contamination, consistent with previous reports (Al-Shehbaz and O’Kane, 2002), the Zn concentrations were >100 μg g^–1^ in soils from 18 sites and >300 μg g^–1^ in those from five sites. This indicated that several sites contained Zn above the critical toxicity of 100–300 μg g^–1^, which would be toxic for most plants.

This surprisingly high Zn concentration in many populations suggest that these Zn may not be of natural origin but affected by human activities. We noticed that about half of these localities are near a human construction (Supplementary Figure 1). Then we classified the localities into near construction or no construction sites. The average Zn concentration in soils at near construction sites was significantly higher than that detected at no construction sites (*P* = 0.00013, Supplementary Table 3 and Figure 1), suggesting artificial introduction of Zn into the habitats where human construction has occurred. Most of the sites with >300 μg g^–1^ Zn in soils were near construction (Supplementary Table 1). However, many sites with no construction also showed considerable levels of Zn (five sites with >100 μg g^–1^ Zn) and are likely to reflect the natural geology (Schlüchter et al., 1981), although it is possible that unobvious previous human activities may have affected, too. These data suggest that the habitat of *A. kamchatica* encompasses soils with high concentrations of Zn due to natural geology or human modification, as well as sites in either category that have low Zn.

The average accumulation of Zn in leaves was 1,416 μg g^–1^ dry weight among 126 plants from the 40 populations. We detected >1,000 μg g^–1^ Zn in 21 of the 40 populations and >3,000 μg g^–1^ Zn in four populations from both near construction and no construction sites (Figure 1 and Supplementary Table 1). The highest leaf concentration of Zn was found in three plants at the Mt. Sirouma site (6,500–6,661 μg g^–1^, Supplementary Table 2) in Japan (2,835 m above sea level), a site near construction with the highest level of Zn in the soil (Figure 1). We found that plants from near construction habitats had, on average, significantly higher quantities of Zn (mean Zn concentration, 1,808 μg g^–1^) than did no construction sites (mean Zn concentration, 926 μg g^–1^) (Supplementary Table 3, *P* = 0.0005, Figure 1). In near construction habitats, there was a significant positive correlation between the leaf and soil concentrations of Zn (*r* = 0.67, *P* < 10^–5^), suggesting that the increased concentrations of Zn observed in the soils at these sites increased the availability of the metal for uptake by plants. In contrast, there was no correlation between the leaf and soil concentrations of Zn in no construction habitats (*r* = −0.007, *P* = 0.95; Supplementary Figure 1).

In the parental species *A. halleri*, collected from two sites in Russia and two sites in Japan, we detected >3,000 μg g^–1^ Zn in the leaves of all four populations, with the highest levels found in plants from the Tada mine site in Japan [average of six replicates = 16,068 μg g^–1^ (Supplementary Table 1)]. Soils were collected at the two mine sites in Japan [Tada and Omoidegawa (OMD)] (Supplementary Table 2), and the Zn concentration (1,317–2,490 μg g^–1^ Zn) was several times higher than at any other site containing *A. kamchatica*.

### Cd and Serpentine Soils in the Habitats of *A. kamchatica*

**FIGURE 2:**
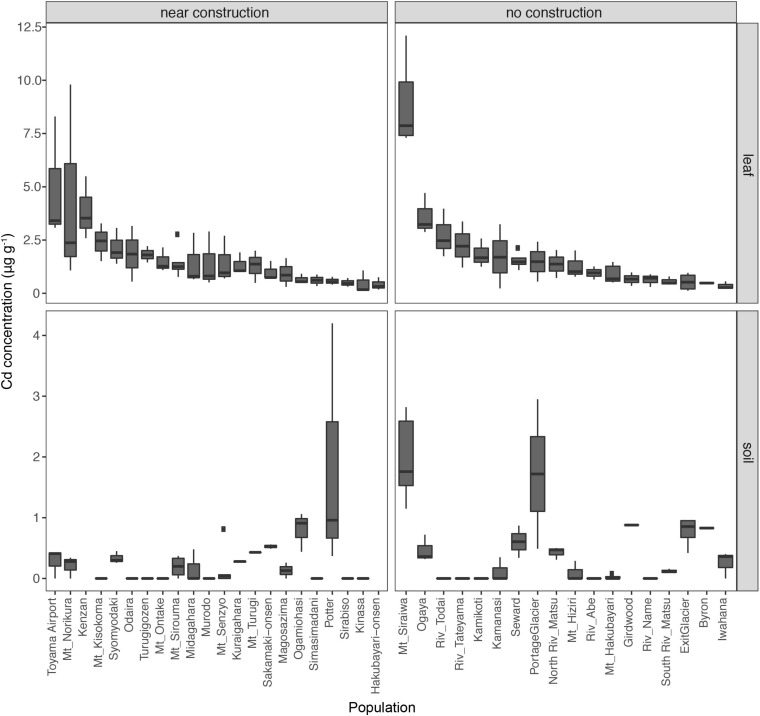
Leaf concentrations of cadmium (Cd) in 40 natural populations of *A. kamchatica* **(Upper)**. Soil concentrations of Cd **(Lower)** at 38 corresponding sites. Populations were grouped into near construction or no construction types. The populations are sorted from highest to lowest Cd concentration in the leaf samples. The *y*-axis is the Cd concentration in μg per gram of dry weight. Note the different *y*-axis scales for leaf and soil samples. Boxplots: center line, median; box limits, upper and lower quartiles; whiskers, 1.5 × interquartile range; points, outliers. The correlations between leaf accumulation and soil concentrations: [near construction Pearson’s *r* = –0.08, (no construction) Pearson’s *r* = 0.58 (see Supplementary Figure 1)]. Refer to Supplementary Table 1 for mean values, variance, number of replicates, and location information.

The soils from all sites had <2 μg g^–1^ Cd, indicating the absence of contamination with this heavy metal in the habitats of *A. kamchatica*. The average accumulation of Cd in leaves among the populations of *A. kamchatica* was 1.8 μg g^–1^ (Figure 2 and Supplementary Table 3) and the Cd concentration in leaves in the five populations with the highest Cd levels ranged from 3.6 to 8.9 μg g^–1^. The maximum concentration of Cd in any single plant was 12.1 μg g^–1^ (Supplementary Tables 2, 3) recorded at the Mt. Siraiwa site, which also had the highest Cd levels in soils. The level of Cd accumulation in leaves was much lower than the threshold defined for Cd hyperaccumulation (>100 μg g^–1^) (Krämer, 2010). Unlike Zn, there was no significant difference in Cd concentration in the soils or leaf tissues between the near construction or no construction habitats (Supplementary Table 3). Moreover, there was no correlation between the leaf and soil concentrations of Cd in the near construction habitats, but there was a positive correlation in the no construction habitats (Supplementary Figure 1). We also found only a weak correlation between Zn and Cd leaf accumulation (*r* = 0.08, *P* = 0.39), likely because of the much lower overall Cd concentration in soils and leaves compared with Zn. In summary, the leaf and soil concentrations of Cd were below the critical toxicity levels for plants and were negligible compared with Zn concentrations in the same populations.

Although the main objective of the soil and leaf sampling of *A. kamchatica* was to quantify the heavy metals Zn and Cd, we also found high levels of magnesium (Mg) and nickel (Ni) in the soils of two populations from Japan (Mt. Sirouma and Mt. Hakubayari) (Supplementary Figures 2, 3 and Supplementary Table 4). These two sites had nearly an order of magnitude greater Mg and Ni levels than those observed for all other populations [the Mt. Sirouma site also contained the highest soil concentration of Zn among all of the *A. kamchatica* sites (643 μg g^–1^)]. High concentrations of Mg and Ni and low calcium-to-magnesium ratios (Ca:Mg) indicate serpentine soils (Kazakou et al., 2008). Consistent with this, the Ca:Mg in soils from Mt. Sirouma (Ca:Mg = 3.71e^–05^) and Mt. Hakubayari (Ca:Mg = 3.45e^–05^) were at least an order of magnitude lower than the average value among all other Japanese populations (on average 1.60e^–03^, sd. 2.60e^–03^). Moreover, the concentrations of Ni in the leaf tissues collected in the Mt. Sirouma, Mt. Hakubayari, and North and South River Matu sites were the highest among all Japanese populations (Supplementary Figure 3), and Mg concentration was highest among the leaf tissues from the Mt. Hakubayari and North and South River Matu populations (Supplementary Figure 2). Therefore, the high levels of Mg and Ni and the low Ca:Mg in soils and leaves at these sites indicate that *A. kamchatica* lives on serpentine soils; in fact, the broader mountain range that includes Mt. Sirouma and Mt. Hakubayari has serpentine soils (Hatano and Matsuzawa, 2008), and the North and South River Matu originates in this mountain range where runoff can deposit Mg and Ni to lower elevations.

### Variation in Zn Accumulation in Experimental Conditions

**FIGURE 3:**
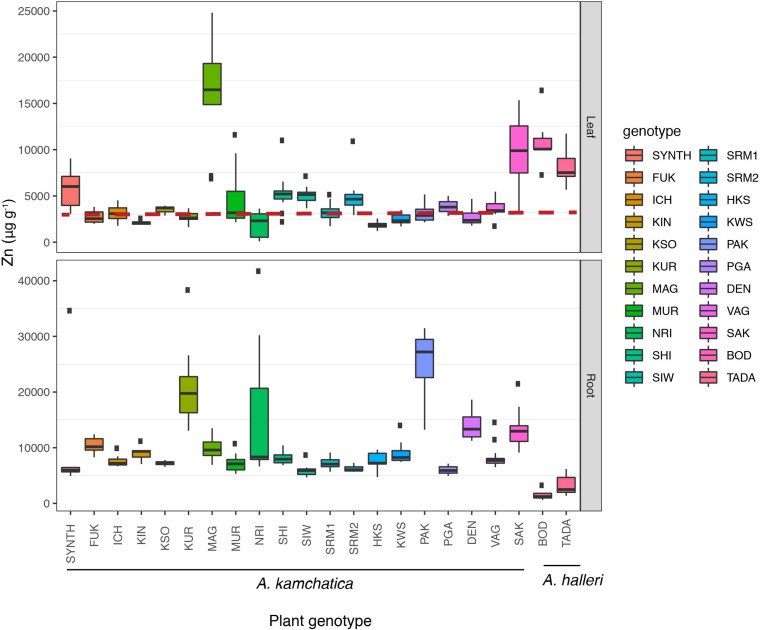
Zinc accumulation in leaf tissues **(Upper)** and root tissues **(Lower)** in 20 *A. kamchatica* and two *A. halleri* genotypes after 1 week of 500 μM zinc treatment in hydroponic chambers. The dashed red line represents 3,000 μg. Boxplots: center line, median; box limits, upper and lower quartiles; whiskers, 1.5 × interquartile range; points, outliers. Boxplots are colored according to plant genotype. Refer to Supplementary Table 5 for mean and median values, standard deviations, and number of replicates.

To examine the quantitative variation of Zn hyperaccumulation in *A. kamchatica* in a common environment, Zn accumulation in leaves and roots was quantified using hydroponic growth chambers. We used a treatment condition of 500 μM Zn for 7 days. Previously, we treated four genotypes of *A. kamchatica*, one *A. halleri* genotype and one *A. lyrata* genotype, and reported that the leaf Zn concentration of *A. kamchatica* was much higher than that of *A. lyrata* but about half of *A. halleri* (Paape et al., 2016). Here we used 20 *A. kamchatica* as well as 2 *A. halleri* genotypes. Among the 20 plant genotypes tested, the average Zn accumulation in leaves was 4,562 μg g^–1^ and the mean values ranged from 1,845 to 16,213 μg g^–1^ among replicates of all genotypes (Figure 3 and Supplementary Table 5A). Linear models detected significant variation in Zn accumulation, even when the outlier genotypes were removed (Supplementary Table 5B). The broad-sense heritability (*H*^2^) for leaf accumulation of Zn in *A. kamchatica* was estimated to be as high as 0.70. In addition, we treated nine of the 20 *A. kamchatica* genotypes with a higher concentration of Zn (1,000 μM) and found significant increases in Zn accumulation in the leaves in all but two genotypes (Supplementary Figure 4). To check whether Zn accumulation among these samples was correlated with the soil concentrations of Zn in our field-collected samples, we compared 9 populations of *A. kamchatica* for which both soil Zn concentration and leaf accumulation in the growth chamber experiment were available. The variance of Zn concentration in leaf tissues that was explained by Zn concentration in soil was not significant (*P* = 0.8); this variance could be explained mostly by plant genotype (*P* < 2e^–16^).

For comparison, we included two *A. halleri* genotypes in the 500 μM Zn experiment. The TADA and BOD genotypes accumulated 8,036 and 10,891 μg g^–1^ Zn in leaf tissues, respectively (Figure 3 and Supplementary Table 5). While the average Zn accumulation in the leaves of *A. kamchatica* is about half of *A. halleri*, two natural genotypes (the MAG and SAK genotypes) accumulated Zn in leaves at similar or higher levels compared with *A. halleri*. A synthetic allopolyploid generated from *A. halleri* ssp. *gemmifera* and Siberian *A. lyrata* ssp. *petrea* (Akama et al., 2014) was included for comparison with natural polyploid genotypes and the TADA genotype. The synthetic polyploid showed a higher level of Zn accumulation in leaves than most natural genotypes (5,845 μg g^–1^). However, this value was significantly lower than the *A. halleri* parental genotype (TADA) (*P* = 0.04, Supplementary Table 6). In contrast to natural *A. kamchatica* which experienced evolutionary changes after polyploid speciation such as mutation and gradual attenuation of expression over time due to environmental conditions, the synthetic allopolyploid provided direct experimental evidence that Zn hyperaccumulation from *A. halleri* can be inherited by *A. kamchatica*, but that the trait is reduced by the allopolyploidization.

The major difference in Zn accumulation between *A. kamchatica* and *A. halleri* was in the roots. The Zn concentrations in the roots of the 20 *A. kamchatica* genotypes were 5,980–25,650 μg g^–1^, whereas Zn concentration in the roots of *A. halleri* was 3,190 μg g^–1^ for the TADA genotype and 1,525 μg g^–1^ for the BOD genotype; this was lower than all *A. kamchatica* genotypes (Figure 3 and Supplementary Table 5A). The shoot/root ratio of Zn accumulation was >1 (i.e., higher Zn concentrations in leaves vs. roots) for both genotypes of *A. halleri*, while all but one of the *A. kamchatica* genotypes had a leaf-to-root ratio of Zn accumulation <1 [the MAG genotype had a leaf-to-root ratio >1 (Supplementary Table 5A)]. Pairwise comparisons of leaf accumulation showed that nearly all *A. kamchatica* genotypes, except MAG and SAK, differed significantly from both *A. halleri* genotypes (Supplementary Table 6A). Using a linear model with Zn accumulation as the dependent variable and species (*A. kamchatica* and *A. halleri*) as the explanatory variable (Supplementary Table 6B), we found that Zn accumulation in leaves was significantly greater in *A. halleri* (*p* = 0.028) and Zn accumulation in roots was significantly greater in *A. kamchatica* (*p* = 0.00067).

**FIGURE 4:**
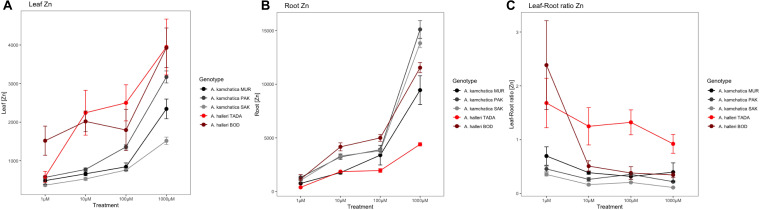
Accumulation of Zn in the leaves **(A)** and roots **(B)** of *A. halleri* and *A. kamchatica* over a 10-fold gradient of Zn concentrations. Points and lines are color coded as follows: *A. halleri* (TADA, red) and three *A. kamchatica* genotypes (PAK, light gray; SAK, dark gray; and MUR, black) at four Zn treatment conditions (1, 10, 100, and 1,000 μM). The ratio of leaf-to-root Zn accumulation at each treatment condition is shown in **(C)**.

The long duration of the 500 μM Zn treatment (1 week) may have resulted in Zn transport approaching equilibrium levels in *A. kamchatica*. With sufficient time, the polyploid could potentially accumulate levels of Zn similar to those of *A. halleri*. To obtain a better picture of the relative efficiency of the physiological transport of Zn from roots to shoots by *A. kamchatica* and *A. halleri*, we measured Zn accumulation over a 10-fold gradient of concentrations within a short time period (48 h). We tested Zn accumulation in the leaves and roots of two *A. halleri* genotype representing a mine-site (TADA) and a non-mine site (BOD) and three *A. kamchatica* genotypes (MUR, PAK, and SAK). In general, the concentration of leaf Zn is lower in *A. kamchatica* than in *A. halleri*, and that of root Zn is higher, although the difference among genotypes were large. The two *A. halleri* genotypes show considerable differences in leaf and root accumulation of Zn, consistent with previous report of differences between mine and non-mine sites (Stein et al., 2017). At basal concentrations of Zn (1 μM), the BOD genotype accumulated significantly more than the TADA genotype (Figure 4A), with more similar levels of accumulation between the two genotypes at higher concentrations. In the roots, the BOD genotype accumulated significantly more Zn at the 10–1,000 μM treatments than TADA (Figure 4B), resulting in a lower shoot-to-root ratio at these treatments (Figure 4C). The shoot-to-root ratio of Zn concentration in each of the three treatment conditions was ≥1 for the TADA *A. halleri* genotype while the ratio is <1 for the BOD genotype (Figure 4C).

Among the three *A. kamchatica* genotypes, we found variation in Zn accumulation following the 10–1,000 μM treatments where the MUR genotype accumulated the highest amount of Zn and the SAK genotype had the lowest accumulation in leaves after the three treatments. Root tissues in these *A. kamchatica* genotypes accumulated Zn at levels between the two *A. halleri* genotypes, except at 1,000 μM where the PAK and SAK genotypes accumulated more Zn than both *A. halleri* genotypes. The high levels of Zn accumulation in the roots of *A. kamchatica* resulted in shoot-to-root ratios ≤0.5 at all Zn concentrations, which was significantly less than the TADA *A. halleri* genotype at each treatment level, but the shoot-to-root ratios are similar to the BOD *A. halleri* genotype (Figure 4C). These comparisons demonstrated that Zn accumulation is leaves of *A. kamchatica* is less than *A. halleri*, but that shoot-to-root ratios are within range of *A. halleri*.

### Homeolog Expression Ratios of Candidate Genes for Heavy Metal Transport

**FIGURE 5:**
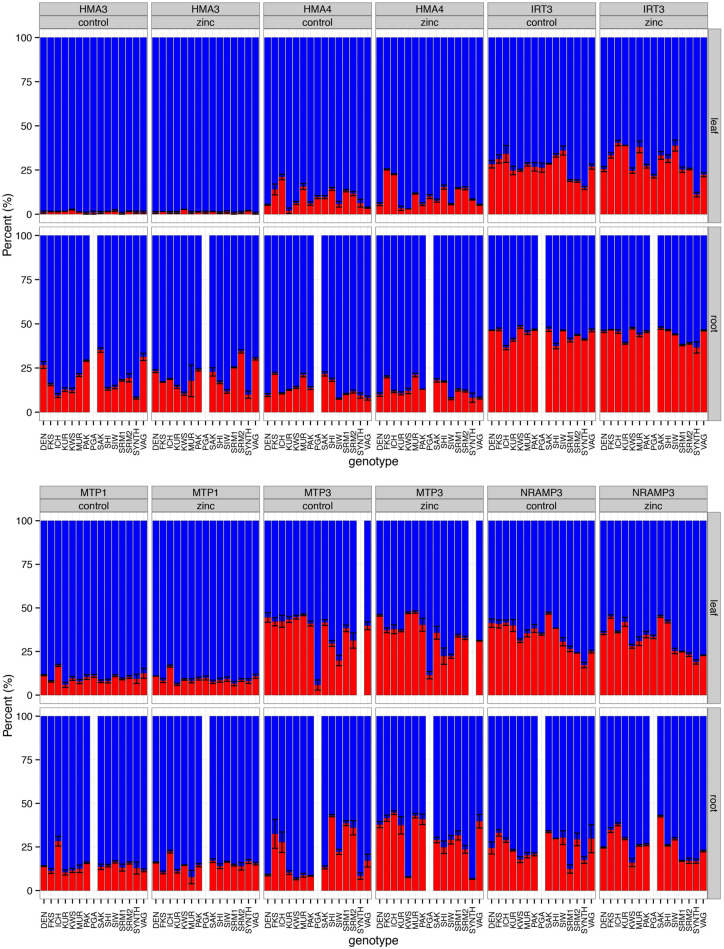
The ratios of homeolog expression for six genes were estimated by pyrosequencing. Each bar on the horizontal axis represents 15 *A. kamchatica* genotypes (14 for root tissues) under control conditions. The vertical axis is the percentage of *A. halleri*-derived homeologs (blue) and *A. lyrata*-derived homeologs (red). For each gene, we estimated expression ratios under control (1 μM Zn) and zinc-treatment (500 μM Zn) conditions; plants were sampled 48 h after the administration of the treatment. Root data were not collected for the PGA genotype (data used to plot bar graphs can be found in Supplementary Table 10).

We found an overall trend toward higher expression of *A. halleri-*derived (H-origin) homeologs compared with the *A. lyrata*-derived (L-origin) homeologs for all of the genes tested, although considerable variation was observed (Figure 5; see Supplementary Table 8 for gene function information). Linear models showed that gene, plant genotype, and tissue contributed to a significant proportion of the variance in expression ratios (*P* < 2.2e^–16^, 3.52e^–13^, and 1.02e^–10^, respectively; Supplementary Table 9). The genes *IRT3*, *MTP3*, and *NRAMP3* exhibited significant variation among the genotypes (*P* = 7.15e^––07^, 3.89e^–14^, and 1.72 e^–11^, respectively), while the *HMA3*, *HMA4*, and *MTP1* genes showed no significant among-genotype variation in expression ratios (*P* = 0.72, 0.72, and 0.21, respectively).

Zinc treatment had no significant effect on gene expression ratios compared with control conditions when both leaf and root tissues were included in the model (treatment *P* = 0.64) or when tissues were analyzed separately (leaf tissue only, *P* = 0.66; root tissue only, *P* = 0.12). These results reflect the constitutive expression of the H-origin metal transporters. Furthermore, we found no significant relationship between homeolog expression ratios and Zn hyperaccumulation levels in our experiments. The model coefficient for the interaction between gene and treatment was non-significant (gene × treatment interaction, *P* = 0.18) and the coefficients for each individual gene and treatment interaction were also non-significant (Supplementary Table 9). The only exception was the *MTP3* gene × treatment × tissue (root) interaction, which exhibited a significant coefficient (*P* = 0.0005). Whether this ratio change was caused by the up-regulation of the L-origin copy or the down-regulation of the H-origin copy cannot be determined by pyrosequencing, but the pattern is consistent with the up-regulation of the L-origin copy observed in a previous RNA-seq experiment (Paape et al., 2016).

## Discussion

### Metalliferous and Non-metalliferous Soils and High Zn Accumulation in the Leaves of *Arabidopsis kamchatica*

In this first species-wide survey of *A. kamchatica*, ion measurements in leaf samples collected from plants growing in natural conditions showed that the species can accumulate large amounts of heavy metals. In particular, Zn accumulation was high in many populations, despite the generally low heavy metal concentrations in soils for the majority of sites. Substantial concentrations of Zn in leaves were found in more than half of the populations (>1,000 μg g^–1^), and plants from four populations had >3,000 μg g^–1^ Zn, a threshold used to define hyperaccumulation in plants growing outside (Krämer, 2010). It is noteworthy that recent experiments have shown that *A. halleri* plants that accumulated up to 1,000 μg g^–1^ of Zn experienced a significant reduction in herbivory compared with plants with lower Zn accumulation (Kazemi-Dinan et al., 2014, 2015). Therefore, a similar level of Zn in leaves could also be sufficient for deterring herbivory in *A. kamchatica*.

Many of the habitats of *A. kamchatica* were metalliferous based on the criteria by Bert et al. (2002) but not due to mining, while the majority would be considered non-metalliferous. This was a major difference from *A. halleri* where many sites were highly contaminated because of mining activities (Pauwels et al., 2006; Hanikenne et al., 2013; Briskine et al., 2017), including two sites containing *A. halleri* used in this study (TADA and OMD). The influence of the *A. lyrata* genome may have reduced the ability of *A. kamchatica* to inhabit highly toxic mine sites. Nevertheless, we found that nearly half of the sites from which samples were collected were clearly modified by human activities such as construction, and the use of corrugated galvanized iron and gratings may have artificially increased Zn levels in the surrounding soils near mountain lodges and roads. Several of these sites contained high concentrations of Zn in the soils, and the Zn concentrations in the leaves near construction were significantly higher than those with no construction. This demonstrated that greater availability of Zn in the soils tends to result in plants that have higher levels of Zn in the leaves as we found in many of the modified sites. It is possible that inheritance of hyper-tolerance to heavy metals from *A. halleri* pre-adapted *A. kamchatica* to expand into human-modified sites that contained elevated Zn in soils.

Compared with Zn, the levels of Cd in soils or in field-collected *A. kamchatica* were unremarkable and below the toxic thresholds. There appeared to be no anthropogenic influence on Cd in the soils, as quantities at both human modified (near construction) and non-modified (no construction) sites differed only slightly and not significantly. Pollution from mining activities is the most common source of high amounts of Cd in soils and plants (Alloway and Steinnes, 1999), and elevated Cd from human construction is not a likely source of increased Cd. The correlation between Zn and Cd concentrations in leaf tissues of *A. kamchatica* plants was low, likely because of a lower exposure of plants to Cd, even among the populations that grew in soils with higher Zn concentrations.

### Experimental Treatments Show That Zn Accumulation Is a Constitutive Trait in *A. kamchatica*

Because the range of concentrations of Zn in field-collected leaf samples varied by more than two orders of magnitude (from 30 to >6,000 μg g^–1^), defining a species as a hyperaccumulator based on a single set threshold may be too simple for the characterization of intraspecific variation from samples collected in natural environments. Therefore, we used experimental treatments to quantify genotypic variation, so that the same exposure to Zn was provided to all plants. Our experiments detected an eightfold difference between genotypes for the lowest and highest Zn accumulation in leaf tissues, indicating a significant variation of Zn hyperaccumulation in *A. kamchatica*. Despite this variation under uniform treatments, the average Zn accumulation in leaves among the *A. kamchatica* genotypes was above 4,000 μg g^–1^. We interpret this as a clear demonstration that *A. kamchatica* has constitutive hyperaccumulation ability as it is understood in the diploid parental species *A. halleri* (Hanikenne et al., 2008; Stein et al., 2017). Further support for constitutive Zn hyperaccumulation may be provided by the lack of a significant relationship between Zn concentrations in native soils and leaf accumulation levels in experimental conditions using *A. kamchatica* germplasm from the sites where soils were collected. Similarly, even with a large sampling of *A. halleri* genotypes from non-metalliferous sites, Stein et al. (2017) found no significant correlation between Zn concentrations in native soils and plants from the sites that were grown experimentally using Zn-amended soils (see Figure 3C in Stein et al., 2017).

We directly tested the inheritance of hyperaccumulation using a synthetic allopolyploid generated from *A. halleri* and *A. lyrata*. The synthetic *A. kamchatica* exhibited 73% of the accumulation of Zn in leaves measured in the parental *A. halleri* strain used in the same experiment (Figure 3 and Supplementary Table 5A). This was considerably higher than that observed for most natural genotypes, which have on average about 50% of the accumulation of Zn in leaves measured in *A. halleri*. This clearly demonstrated that hyperaccumulation can be retained after hybridization between divergent parental species, although the *A. lyrata* genome had a weakening effect (by 27%) on this trait when Zn accumulation in *A. kamchatica* is compared with *A. halleri*.

Testing Zn accumulation in leaf and root tissues over a gradient of Zn treatments allowed us to further examine the physiological capacity of Zn accumulation in *A. kamchatica* compared with the parent species *A. halleri*. This experiment demonstrated that the shoot-to-root ratio in three polyploid genotypes never exceeded 0.5 at any treatment, while the shoot-to-root ratio in *A. halleri* for the TADA genotype that originated in a toxic mine site in Japan was ≥1 at all treatments. By contrast, the BOD genotype showed a strong hyperaccumulation response in leaves at the 1 μM Zn treatment but had similarly low accumulation in roots compared to all other genotypes including the TADA *A. halleri* genotype, but had a more similar shoot-to-root ratio to *A. kamchatica* (<1) at the 10–1000 μM treatments. A similar experiment was conducted comparing *A. halleri* and *A. thaliana* (a non-hyperaccumulator) (Talke et al., 2006), where the shoot-to-root ratio in *A. halleri* was similar to our results, but the shoot-to-root ratio (∼0.05) in *A. thaliana* was an order of magnitude lower compared with the shoot-to-root ratio we measured in *A. kamchatica* in this study.

We propose two explanations for the reduction in heavy metal hyperaccumulation in *A. kamchatica* compared with the diploid hyperaccumulator parent *A. halleri*. First, allopolyploidization is expected to reduce or attenuate the net expression levels of metal transporters compared with the diploid parents. Because allopolyploidization results in a state of fixed heterozygosity of functionally duplicated gene copies (homeologs), expression of homeologs may be reduced compared with the diploid parents. Previously, RNA-seq analysis showed a reduction of ∼50% in the expression of several genes encoding heavy metal transporters was detected in the *A. halleri*-derived homeologs of *A. kamchatica* compared with the orthologous genes in *A. halleri* (Paape et al., 2016). This reduction in the expression levels of metal transporter genes was consistent with the ∼50% lower levels of Zn accumulation in the leaves of natural accessions of *A. kamchatica* compared with *A. halleri*. Fixed heterozygosity can result in a trait that resembles a balanced polymorphism with a semi-intermediate phenotype. It is important to note that although the hyperaccumulation trait in *A. kamchatica* was reduced by ∼50% compared with *A. halleri*, it was an order of magnitude greater than that of the non-hyperaccumulating parent *A. lyrata* (Paape et al., 2016). Second, inhibiting genetic factors derived from the *A. lyrata* parental genome likely contribute to the reduced phenotype in the polyploid compared with *A. halleri*. It is expected that these genetic factors prevent the transport of toxic heavy metals to leaf tissues, which limits their toxicity in leaves. This is an example of genomic antagonism resulting from the divergent parental genomes.

### Expression Bias in *A. halleri*-Derived Homeologs

Because Zn hyperaccumulation was inherited from *A. halleri*, we expected that the homeolog expression ratios would show a pattern consistent with a parental legacy effect of gene expression (Buggs et al., 2014). Pyrosequencing revealed that the expression ratios of homeologs for six genes with roles in heavy metal hyperaccumulation or metal tolerance exhibited higher expression of the H-origin copy. This suggests that homeolog-specific expression is maintained by *cis*-regulatory differences (Shi et al., 2012; Yoo et al., 2014). The two main loci involved in heavy metal hyperaccumulation in *A. halleri* are the heavy metal ATPase 4 (*HMA4*) gene, which encodes an ATPase transporter protein, and the metal tolerance protein 1 (*MTP1*; also called *ATCDF1* or *ZAT1*) gene, which encodes a cation diffusion facilitator (CDF) protein (Frérot et al., 2010). In *A. halleri*, *HMA4* is tandemly triplicated and *MTP1* has at least three copies (Hanikenne et al., 2008; Shahzad et al., 2010; Briskine et al., 2017). A single copy of these two genes is present in *A. lyrata* (Briskine et al., 2017; Paape et al., 2018). Both genes have significantly higher expression than their non-hyperaccumulating congeners because of *cis*-regulation and gene duplications (Hanikenne et al., 2008; Shahzad et al., 2010). The *HMA3* gene (which encodes an ATPase in the same class as *HMA4*) also has a putative role in the vacuolar sequestration of Zn (Morel et al., 2008), similar to *MTP1*, but is only found in a single copy in both *A. halleri* and *A. lyrata* (Paape et al., 2018). These features are consistent with constitutive gene expression inherited from the hyperaccumulating diploid parent, *A. halleri*, which would be essential for retaining the hyperaccumulation phenotype in the species-wide collection of *A. kamchatica* examined in this study.

The ZIP-transporter *IRT3* and natural-resistance-associated macrophage protein 3 (*NRAMP3*) genes encode iron (Fe) transporters that are coregulated by Fe and Zn and show significant expression differences between the hyperaccumulator *A. halleri* and the non-accumulators *A. thaliana* (Talke et al., 2006; Lin et al., 2009; Shanmugam et al., 2011) and *A. lyrata* (Filatov et al., 2006; Paape et al., 2016). The *IRT3* and *NRAMP3* homeologs also exhibited an H-origin expression bias, which was consistent with previous differential expression studies that compared *A. halleri* with *A. lyrata* or *A. thaliana* in Zn-treatment studies (Filatov et al., 2006; Talke et al., 2006); however, their direct role in Zn hyperaccumulation is less clear (Thomine et al., 2003). Moreover, both *IRT3* and *NRAMP3* showed a much larger variation in expression ratio compared with *HMA4* and *MTP1*, which may reflect a greater constraint on the constitutive expression of the latter two genes.

Most importantly, Zn treatment had no significant effect on the expression ratio of five of these six genes, demonstrating the *A. halleri*-derived constitutive expression of genes with known or putative roles in Zn hyperaccumulation. The gene *MTP3* was an exception, as it exhibited a significant change in homeolog ratios in the root tissues of many genotypes after Zn treatment. This expression-ratio change was most likely the result of the previously demonstrated upregulation of the L-origin homeolog in roots of one of the genotypes used in the current study (Paape et al., 2016). *MTP3* prevents heavy metal transport to the shoots by sequestering Zn in the vacuoles in the roots of *A. thaliana* (Arrivault et al., 2006). We assume that this gene plays a similar role in *A. lyrata*, and is therefore a potential *A. lyrata*-derived inhibiting factor that would contribute to the reduced leaf hyperaccumulation observed in *A. kamchatica*.

### Evolutionary Scenario of an Allopolyploid Habitat Expansion

Adaptability and range expansion in polyploids have been debated for several decades (Stebbins, 1971; Soltis et al., 2014; Van de Peer et al., 2017). However, empirical examples of genetically tractable quantitative traits that have ecological relevance are lacking (Godfree et al., 2017). We suggest that the inheritance of hyperaccumulation from *A. halleri* conferred advantages instantaneously following polyploid speciation, which was estimated to have occurred ∼100,000 years ago (Paape et al., 2018), according to the following scenario. First, *A. kamchatica* became tolerant to soils with toxic levels of heavy metals that were present in the areas of growth of natural populations because of geological processes. Subsequently, during the past few thousand years, soils became contaminated by human activities, and the tolerance functioned as a pre-adaptation to modified environments (as proposed for *A. halleri*; Meyer et al., 2016). In contrast to *A. halleri*, and consistent with its attenuated Zn hyperaccumulation, *A. kamchatica* was not found in extremely contaminated sites, such as mines. We found a distinct, intermediate habitat and species distribution of *A. kamchatica* such as soils near mountain lodges and roads. This supports the importance of a fine-scale environment for the habitat differentiation of polyploid species (Akiyama et al., 2019) in addition to climatic gradients at a large geographic scale (Hoffmann, 2005). Furthermore, Zn concentration in the leaves of the majority of the natural *A. kamchatica* populations was >1,000 μg g^–1^, which was an effective level for insect defense in *A. halleri* (Kazemi-Dinan et al., 2014). In addition, we found *A. kamchatica* living on serpentine soils, which has also been reported for the other diploid parent, *A. lyrata* (Turner et al., 2010; Arnold et al., 2016). We hypothesize that *A. kamchatica* expanded its habitats by combining the heavy metal and serpentine tolerances from *A. halleri* and *A. lyrata*, respectively. Our study represents a promising example of the contribution of the inheritance of genetic toolkits for soil adaptation to the habitat expansion of an allopolyploid species. New genomic and transcriptomic capabilities in *A. kamchatica* combined with functional genetics (Yew et al., 2018) and self-compatibility (Tsuchimatsu et al., 2012) provide a unique opportunity to study the genetics of the edaphic and climatic adaptation of a polyploid species (Shimizu et al., 2011).

## Data Availability Statement

All datasets used in this study are included in this study are included in the accompanying Supplementary Files.

## Author Contributions

TP and KS conceived the study. TP and TC designed and performed the experiments and prepared samples for analyses. TP designed the pyrosequencing assays and TC performed the pyrosequencing experiments. TP, YO, AH, TK, and KS collected field samples. TP and TC constructed the datasets. TP and RA performed the statistical analyses. TP drafted the manuscript. TP, RA, and KS revised the final draft. All authors contributed to the article and approved the submitted version.

## Conflict of Interest

The authors declare that the research was conducted in the absence of any commercial or financial relationships that could be construed as a potential conflict of interest.
