# Supplementary material for: Experimental and Field Data Support Range Expansion in an Allopolyploid Arabidopsis Owing to Parental Legacy of Heavy Metal Hyperaccumulation
Source: Front Genet. 2020 Sep 30;11:565854. doi: 10.3389/fgene.2020.565854 (PMC7554548; doi:10.3389/fgene.2020.565854)
Supplement: Supplementary file 1 [file Data_Sheet_1.PDF]

Supplementary Material

Supplementary Figures

a)

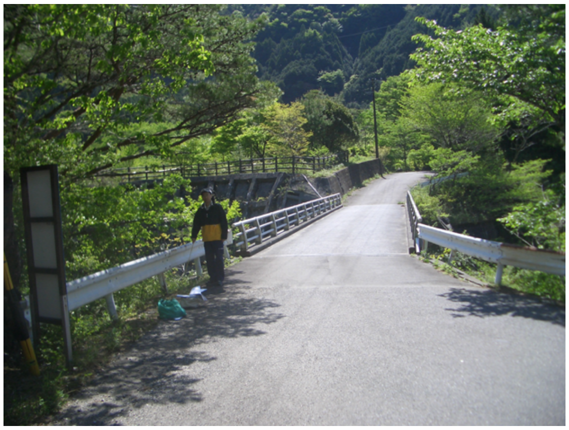

b)

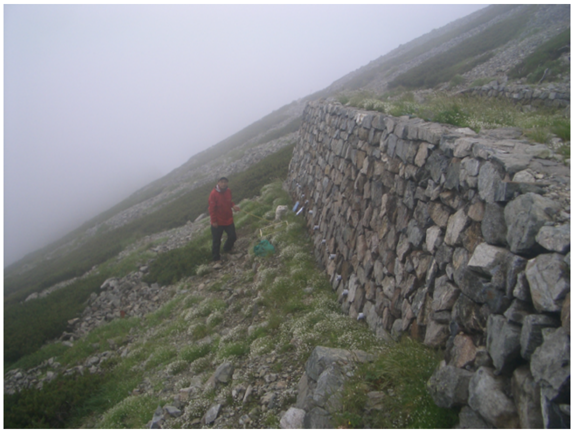

c)

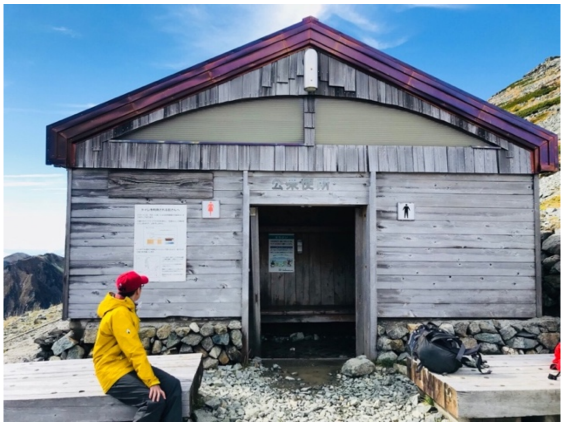

d)

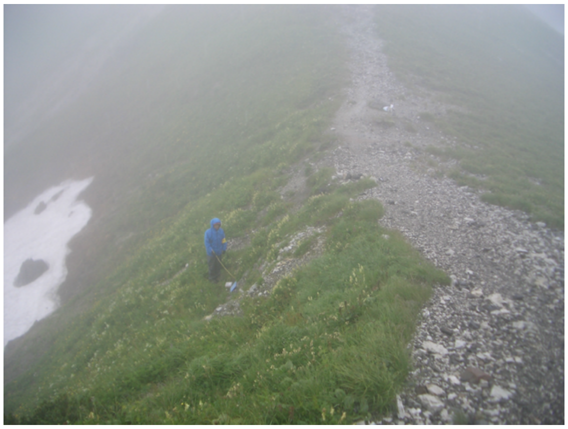

e)

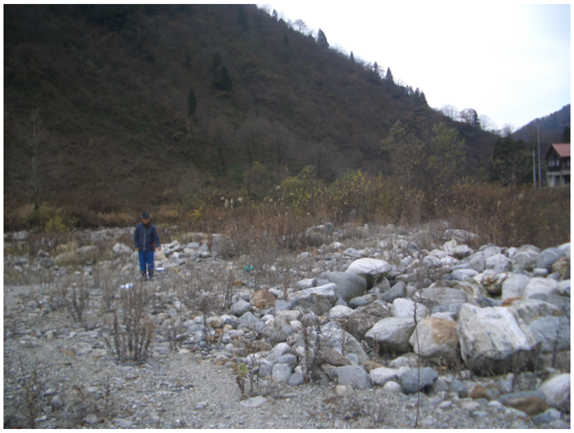

Supplementary Figure S1. Photographs of some of locations were near artificial construction such as a paved road in Magosajima (a) or a mountain lodge in Turugigozen (b, c). Some other examples of locations that were not near artificial constructions include a mountain ridge in Mt. Hakubayari (d) or a riverside in Riv. Tateyama (e). In a,b,d,e, the investigator points to focal plants.

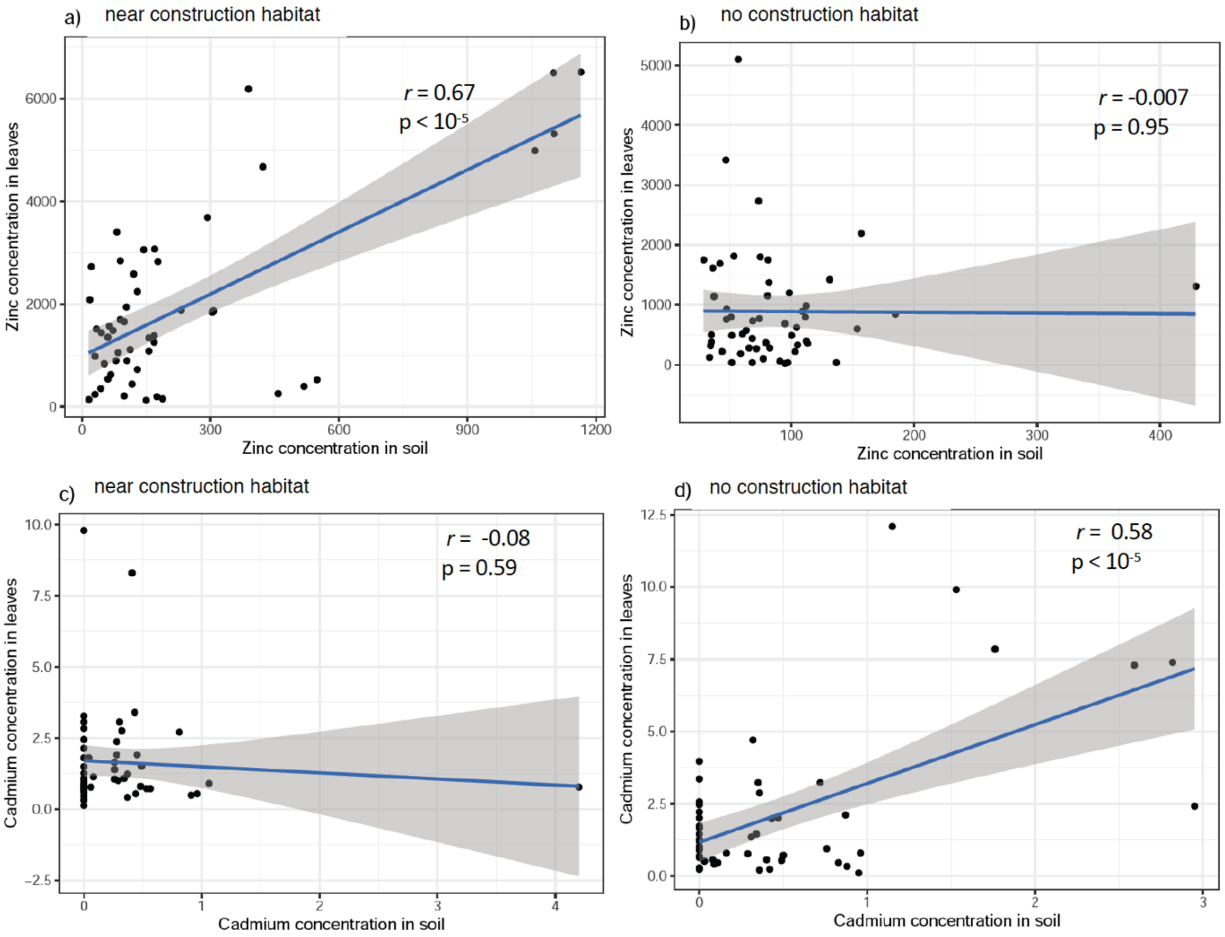

Supplementary Figure S2. Correlations of leaf accumulation and soil concentrations for Zn (a) near construction and (b) no construction habitats. Correlations of leaf accumulation and soil concentrations for Cd (c) near construction and (d) no construction habitats. Pearson's correlation coefficients (*r*) and p-values shown in each panel.

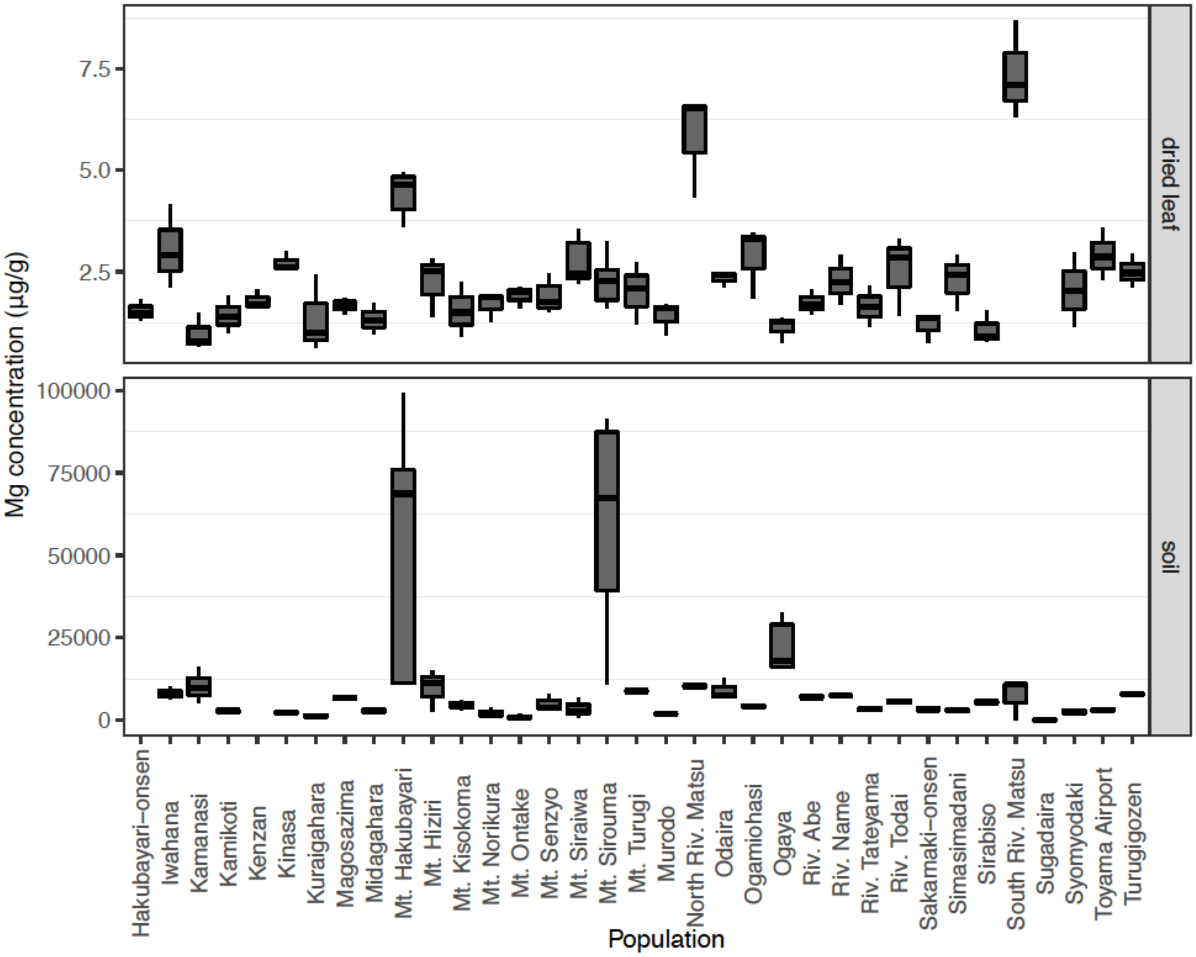

Supplementary Figure S3. Magnesium (Mg) concentrations in leaf tissues (upper panel) and Mg concentrations in soils at each collection site. Boxplots show center line: median; box limits: upper and lower quartiles; whiskers: 1.5 × interquartile range; points: outliers.

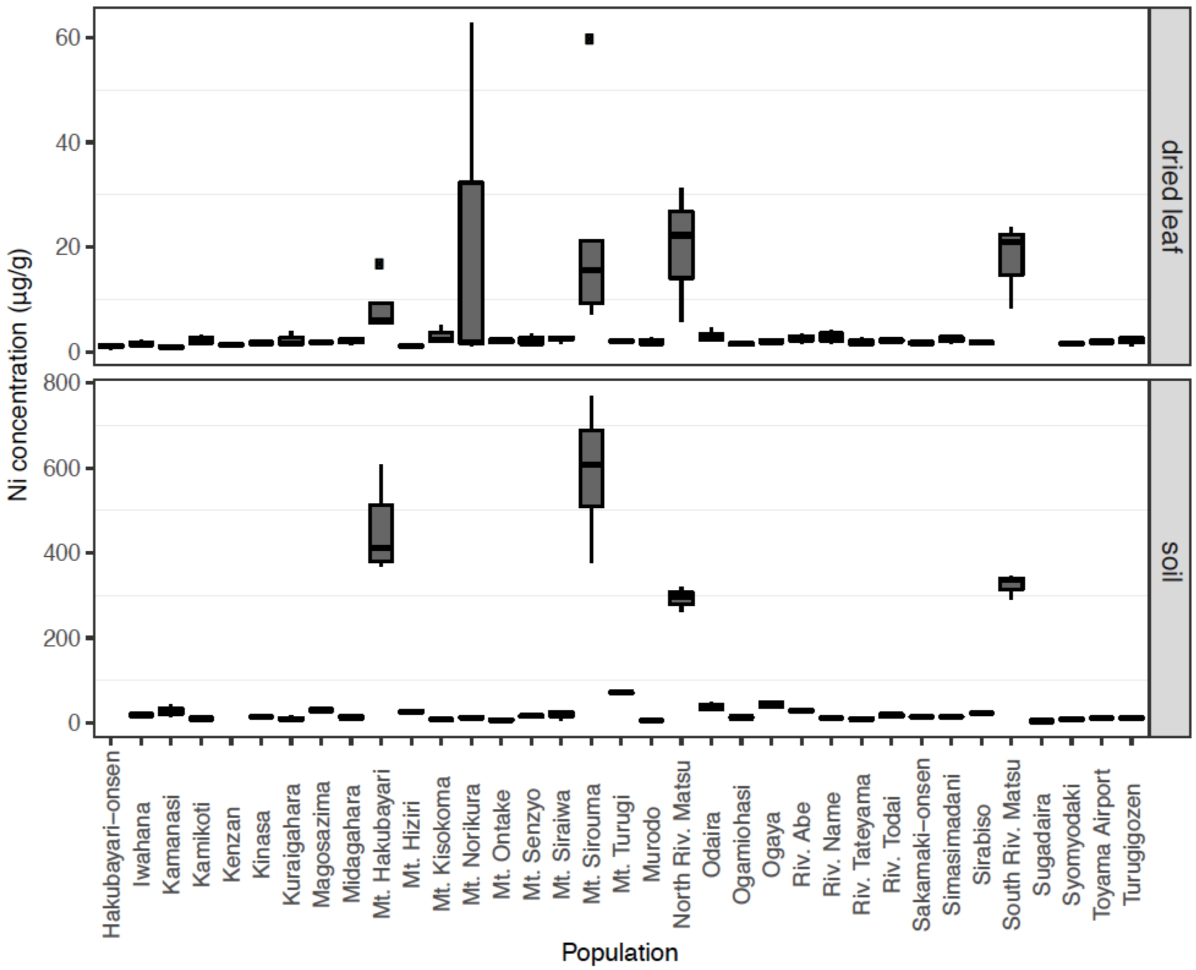

Supplementary Figure S4. Nickel (Ni) concentrations in leaf tissues (upper panel) and Ni concentrations in soils at each collection site. Boxplots show center line: median; box limits: upper and lower quartiles; whiskers: 1.5 × interquartile range; points: outliers.

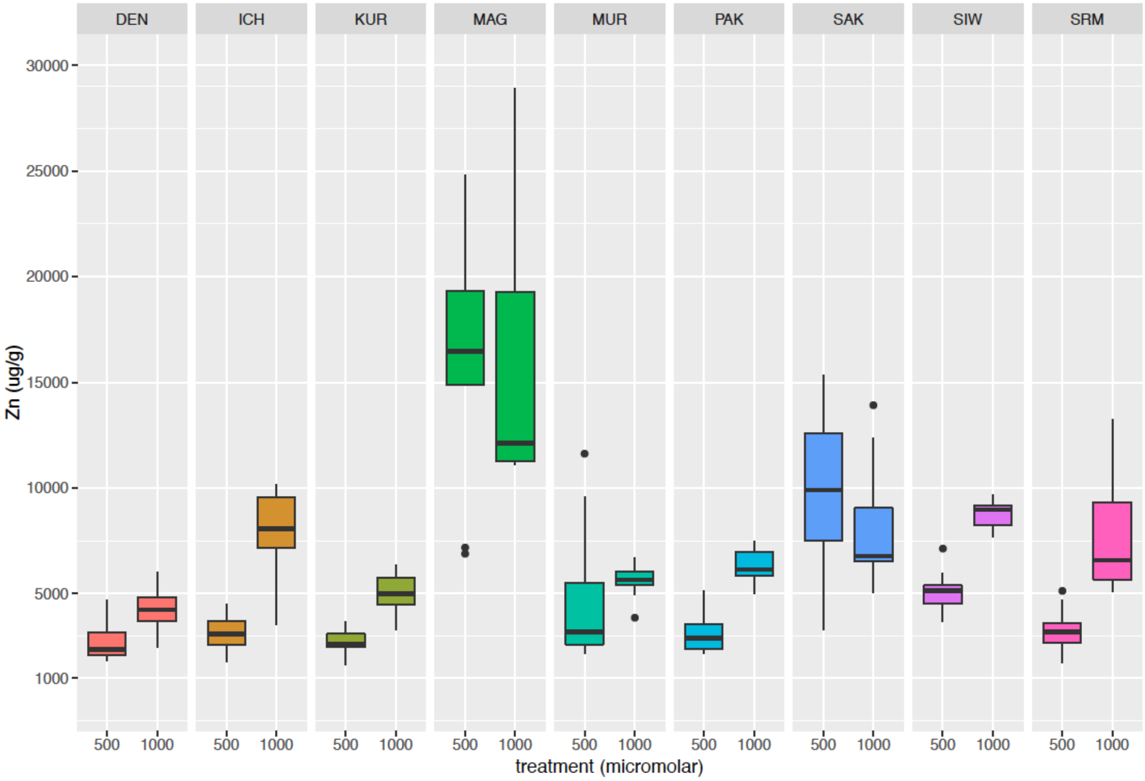

Supplementary Figure S5. Zn accumulation in leaves of nine *A. kamchatica* accessions following 7 days of Zn treatments of 500 and 1000 µM of Zn in two independent experiments (the same plants were not exposed to both 500 and 1000 µM treatment conditions but were grown in separate hydroponic containers in either treatment). Significant increases in mean Zn accumulation were observed in the 1000 µM treatment compared with the 500 µM treatment for all except the MAG and SAK genotypes. The MAG and SAK genotypes showed no significant increase in mean Zn accumulation between 500 and 1000 µM treatments. The two genotypes (MAG and SAK) that showed the highest Zn accumulation in the 500 µM treatment, did not significantly increase Zn accumulation in leaves when treated with 1,000 µM of Zn. This demonstrated that Zn accumulation in *A. kamchatica* leaf tissues may be increased by exposure to higher heavy metals in the roots, but for the two highest accumulating genotypes, it may suggest a maximum threshold or saturation of heavy metals in the leaves under high Zn dosage.
